# Validity of social media for assessing treatment patterns in oncology patients: a case study in melanoma

**DOI:** 10.1093/jamiaopen/ooz013

**Published:** 2019-09-03

**Authors:** Laura McDonald, Varun Behl, Vijayarakhavan Sundar, Faisal Mehmud, Bill Malcolm, Sreeram Ramagopalan

**Affiliations:** 1 Centre for Observational Research and Data Sciences, Bristol-Myers Squibb, Uxbridge, UK; 2 Mu-Sigma, Bengaluru, India; 3 Bristol-Myers Squibb, Uxbridge, UK

**Keywords:** social media, treatment patterns, melanoma, flatiron, MarketScan, McKesson, EMR, claims, validation

## Abstract

There is a need to understand how patients are managed in the real world to better understand disease burden and unmet need. Traditional approaches to gather these data include the use of electronic medical record (EMR) or claims databases; however, in many cases data access policies prevent rapid insight gathering. Social media may provide a potential source of real-world data to assess treatment patterns, but the limitations and biases of doing so have not yet been evaluated. Here, we assessed whether patient treatment patterns extracted from publicly available patient forums compare to results from more traditional EMR and claims databases. We observed that the 95% confidence intervals of proportions of treatments received at first, second, and third line for advanced/metastatic melanoma generated from unstructured social media data overlapped with 95% confidence intervals from proportions obtained from 1 or more traditional EMR/Claims databases. Social media may offer a valid data option to understand treatment patterns in the real world.

## INTRODUCTION

Despite the availability of international and national treatment guidelines for many diseases, these are often not adhered to as a result of patient and physician characteristics and preferences as well as clinical equipoise.^1^ As such, it is important to understand how patients are managed in the “real world” (ie, actual clinical practice) in order to assess the unmet clinical challenges of physicians and needs of patients.^1–3^ Typically, these data are obtained through databases such as electronic medical records (EMRs) or administrative health insurance claims data, or through a form of *de novo* data collection (eg, a medical chart review or a physician survey).^4^^,^^5^ Depending on the approach used, several logistical hurdles may exist including restriction on data access and its cost and lengthy timelines for data collection.^5^

Social media, which includes online forums (eg, Cancer Compass), blogs, microblogs (eg, Twitter), video-sharing (eg, YouTube), and more direct networking sites (eg, Facebook or Patients Like Me), allow users to create and exchange information.^2^ Posted content, even on sites not created exclusively for patients, often include information related to health.^2^ Among hospital patients consenting to provide details of social media accounts for example, 7.5% of retrieved Facebook posts were related to health.^6^ With automated approaches such as natural language processing and machine learning the analysis of hundreds of thousands of text-based posts is now possible allowing for accelerated and more cost-effective data acquisition and analysis as compared to more traditional approaches. One of the main strengths of using social media in biomedical research is that it is void of the Hawthorne effect. This is when patients that are asked to self-report their experiences either through a survey or interview, respondents may be influenced (even unconsciously) by the setting and tend to report what they think is the “best” perceived or expected opinion, rather than a genuine one.^7^ Social media allows researchers to use secondary data to investigate opinions, which is an unprecedented opportunity, at least in biomedical research. As social media offers the potential to generate rapid insight into real-world treatment patterns in a potentially larger and more diverse population than more traditional methods, it is of considerable interest to the research community.^2^ To the best of our knowledge, there is only one previous study assessing treatment patterns from patient forums. This was a text based filtering approach for patient posts in multiple sclerosis.^8^ However, whether or not these treatment patterns were similar to those seen using more traditional methods was not assessed. There are important potential methodological limitations to consider when assessing data from social media, including selection and information bias.^2^ To manage these limitations, it is important to assess the extent of any bias to determine the quality of the health data collected through social media. In this study we therefore sought to compare treatment patterns (ie, understanding what treatments patients are receiving at first, second, and third line) for advanced melanoma obtained through social media with those obtained from EMR and administrative claims databases in the United States.

## METHODS

### Social media data

To gather treatment-related information for melanoma, unstructured text containing patient posts, username, and date of post was scraped from publicly available forums. Data extraction using web scraping is a quicker alternative to track patient treatment than accessing and processing EMR/claims data. All patient posts between the start of 2011 to the end of 2017 were extracted from four publicly available patient forums Melanoma.org^9^ Melanoma International,^10^ Cancer Compass,^11^ and the Cancer Survival Network.^12^ These US-based websites provide a forum for melanoma patients to talk about their disease and treatments. They were selected from a Google search for melanoma patient forums using key search terms such as: “melanoma discussion board,” “melanoma patients’ forum,” and “melanoma discussion forum.” Only sites in the English language and openly accessible were selected. Melanoma.org is created by the Melanoma Research Foundation which is the largest independent nonprofit organization for melanoma patients. Melanoma international is a community established in 2003 for individuals to find out more about risk of melanoma and share their experience and treatment journey. Cancer Compass is a US-based cancer community with over 60 000 patients reporting and posting about their cancer journey. Cancer compass offers separate discussion tabs for each cancer. We focused on the “Melanoma” discussion page to extract data related to melanoma. The Cancer Survival network is an American Cancer Society forum hosting discussions on a number of cancers. The “melanoma” discussion tab was the focus of data extraction from this site.

In order to remove noise, user posts were filtered to those users having at least one post mentioning a treatment. The list of treatments is shown in Supplementary Table S1 and was derived from the National Comprehensive Cancer Network (NCCN) guidelines. Potential typographical errors, spelling mistakes, abbreviations, and brand/generic names for treatments were manually defined (Supplementary Table S2), using a list of all these instances which were extracted from the dataset. As interferon and docetaxel can potentially be used as adjuvant therapy, these were not considered as lines of treatment if received as monotherapy. The posts were further processed to enable analysis including text cleaning (eg, removing URLs, special characters) and broken down to smaller sentences using sentence tokenizing. This enabled the creation of an analytical data file with each cleaned and tokenized sentence tagged to a cus-tom generated user ID (based on username and patient forum and reflecting unique patients) and post date. For each user ID, posts were sorted based on posting date, starting from the earliest to the most recent.

An automated process was developed to classify treatment patterns from the posts. Firstly, a random selection of 7422 posts were manually curated to assess whether they contained information relating to a patient taking a treatment or not. These 7422 manually curated posts were then chosen to create an unbiased dataset for training and testing using a supervised text classification approach. Simple balancing checks were performed to make sure that the random selection was not biased towards one of the classes. Three supervised machine learning algorithms were tested (support vector machine [SVM], naïve Bayes, and K-nearest neighbor) for classification. These approaches are classically used in text classification.^13–15^ TF-IDF (Term Frequency—Inverse Document Frequency) vectorizer was used to generate features. TF-IDF assigns weights to each word, which is considered as a data point for the model. This weight is a statistical measure used to evaluate how important a word is to a document in a corpus. SVM (Linear kernel) showed the best performance for classification (precision 84%; recall 84%) as compared to naïve Bayes (precision 81%; recall 81%) and K-nearest neighbor (precision 68%; recall 50%) with *K* = 5, and therefore, SVM was used to identify the remaining (non-manually curated) posts containing treatment information. The precision values were formulated by measuring the ratio of posts correctly identified as treatment containing compared to all posts predicted to be treatment containing, whereas recall refers to the ratio of posts correctly identified as treatment containing compared to all posts containing treatment information.

CTakes,^16^ negtools,^17^ and SUTime^18^ were then applied to filter out posts where treatments were discussed in the context of not being received (eg, “I did not take temozolomide”) and to provide temporal context to when treatments were received to determine lines of treatment in patients (eg, converting I received temozolomide three months ago into a specific date).

### EMR and claims data

Flatiron, IQVIA, McKesson, and MarketScan are databases frequently used for generating real-world insights into treatment patterns in oncology and were therefore chosen for comparison.

### Flatiron

Data from the Flatiron Health longitudinal EMR database were used for this study. Flatiron covers over 190 cancer clinics in the United States. Patients were selected from the database based on having a diagnosis of advanced or metastatic melanoma and at least two visits following diagnosis at a Flatiron treatment center January 1, 2011–December 31, 2017. Information on anticancer treatments given after advanced/metastatic disease diagnosis and line of treatment as per Flatiron definitions was extracted for analysis.^19^

### McKesson

McKesson Specialty Health maintains iKnowMed, an integrated web-based database and oncology-specific EMR system that captures outpatient practice encounter histories from network community oncology practices affiliated with over 1000 physicians in the US. Patients were selected from the database based on having a diagnosis of Stage III, IIIA, IIIB, IIIC, and IV “Melanoma, Cutaneous” between January 1, 2011 and December 31, 2017. Information on anticancer treatments given after advanced/metastatic disease diagnosis and line of treatment as per McKesson definitions was extracted for analysis.

### Truven health MarketScan

This study used the Truven Health MarketScan Research databases (the Commercial Claims and Encounters database, and the Medicare Supplemental and Coordination of Benefits database); these databases contain claims from employers, health plans, and public organizations. The data in the database comprise service-level claims for inpatient and outpatient healthcare services and outpatient prescription drugs. All US census regions are represented in the databases. Patients were selected from the database based on having a diagnosis of advanced and metastatic melanoma (a diagnosis for metastases on or after the first diagnosis of Melanoma) using international classification of disease (ICD)-9 and ICD-10 codes between January 1, 2011 and December 31, 2017. Information on anticancer treatments given after advanced/metastatic disease diagnosis was extracted and line of treatment was defined using an algorithm aligned to the Flatiron approach.

### IQVIA PharMetrics+

Data from the IQVIA PharMetrics+ Claims Database was used in this study. This database contains adjudicated claims for more than 150 million unique patients across the United States, with diverse representations of geography, employers, payers, providers, and therapy areas.

Patients were selected from the database based on having a diagnosis of advanced and metastatic melanoma (a diagnosis for metastases after the first diagnosis of Melanoma) using ICD-9 and ICD-10 codes between January 1, 2011 and December 31, 2017. Information on anticancer treatments given after advanced/metastatic disease diagnosis was extracted and line of treatment was defined using an algorithm aligned to the Flatiron approach.

### Patient matching

As new treatments are launched, treatments patients will receive will naturally change over time. In order to allow contemporaneous comparisons, patient matching was undertaken between social media and EMR/claims databases. Frequency matching with respect to first line treatment initiation year was performed, with up to 4 patients in the EMR/Claims databases matched to every patient in the social media data. Where patients could not be matched they were dropped.

### Statistical analysis

Data extraction and text processing was performed using Python (Jupyter Notebook) version 2.0. Machine learning analysis was performed using Python libraries (SKlearn/Sci-Kit Learn). Line of treatment analysis for MarketScan, Flatiron, McKesson, and PharMetrics+ was performed using SAS (version 9.4). Comparability of social media estimates with those from databases was investigated by looking at overlap of 95% confidence intervals.

## RESULTS

A total of 107 248 posts were extracted from the cancer forums, which after filtering out for at least one systemic drug mention and text cleaning provided a total of 28 801 posts for further analysis. The majority of these came from Melanoma.org (23 064), with 5188 posts coming from Melanoma International and smaller numbers from Cancer Compass (414) and Cancer Survivor Network (135). In total these posts represented 2828 unique patients. Further processing of these posts to identify which ones contained information on a patient receiving a treatment provided 1196 patients available for analysis of treatment patterns. After matching to patients in the databases which resulted in patient loss due to inability to match, 817 patients from social media, 2666 patients from Flatiron, 3980 patients from MarketScan, 560 patients from McKesson, and 739 patients from PharMetrics+ were included in the analysis. The distribution of patients by year of initiation of first line treatment is shown in Table 1 and the number of patients providing data by treatment line is shown in Table 2.

**Table 1.** Year of first line treatment initiation in each database

Database	Flatiron (*N* = 2666)		MarketScan (*N* = 3268)		McKesson (*N* = 560)		PharMetrics+ (*N* = 739)		Social media (*N* = 817)	
Year	*N*	%	*N*	%	*N*	%	*N*	%	*N*	%
2011	104	3.9	160	4.9	10	1.8	28	3.8	40	4.9
2012	233	8.7	384	11.8	24	4.3	44	6.0	96	11.8
2013	366	13.7	560	17.1	35	6.3	126	17.1	140	17.1
2014	519	19.5	720	22.0	130	23.2	180	24.4	180	22.0
2015	508	19.1	508	15.5	127	22.7	127	17.2	127	15.5
2016	508	19.1	508	15.5	127	22.7	127	17.2	127	15.5
2017	428	16.1	428	13.1	107	19.1	107	14.5	107	13.1

**Table 2.** Number of patients with data on first, second, and third line treatment

Line number	Flatiron		MarketScan		McKesson		PharMetrics+		Social media	
	*N*	%	*N*	%	*N*	%	*N*	%	*N*	%
First	2666	100.0	3268	100.0	560	100.0	739	100.0	817	100.0
Second	930	34.9	1108	33.9	260	46.4	339	45.9	226	27.7
Third	326	12.2	514	15.7	66	11.8	154	20.8	73	8.9

**Figure 1.**
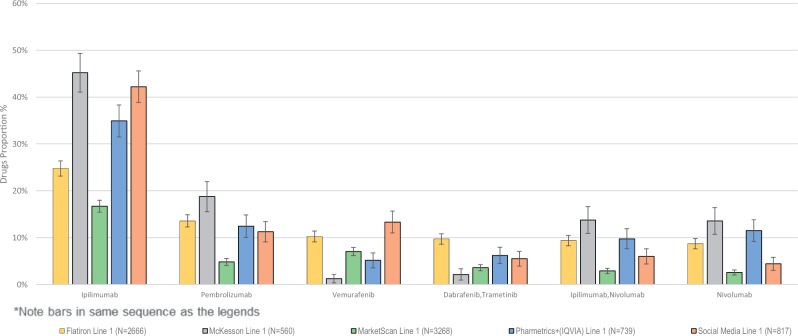
First line treatment proportions and associated confidence intervals.

**Figure 2.**
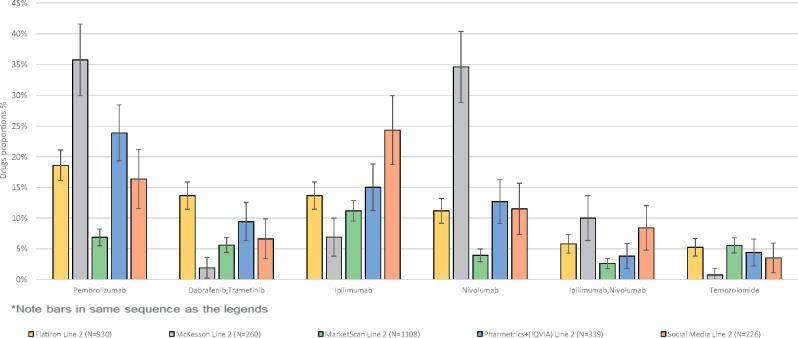
Second line treatment proportions and associated confidence intervals.

**Figure 3.**
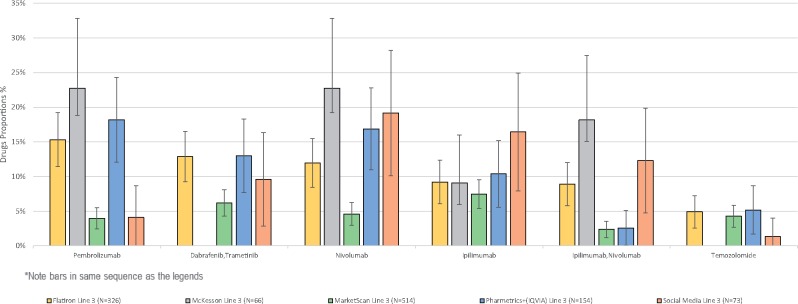
Third line treatment proportions and associated confidence intervals.

Proportions of patients taking treatments at each line are shown in Tables 3–5 and Figures 1–3. For example, in Table 3, the frequency of and associated 95% confidence intervals for the most common treatments (or treatment combinations) given to patients at first line is described. Ipilimumab is the most common treatment given at first line in all databases as well as in the social media data, however the frequency reported in the databases varies from 16.7% (MarketScan) to 45.2% (McKesson). The tables are ranked according to frequency seen in the Flatiron database but this is not necessarily consistent across databases (eg, in Table 4 the combination ipilimumab/nivolumab is the fifth most common treatment at second line in Flatiron but the third most common in the McKesson database).

**Table 3.** First line treatment proportions and associated confidence intervals

Line 1 treatment	Flatiron drug proportion ± confidence interval	McKesson drug proportion ± confidence interval	MarketScan drug proportion ± confidence interval	PharMetrics+ drug proportion ± confidence interval	Social media drug proportion ± confidence interval
Ipilimumab	24.8% ± 1.6%	**45.2% ± 4.1%**	16.7% ± 1.3%	34.9% ± 3.4%	42.2% ± 3.4%
Pembrolizumab	**13.6% ± 1.3%**	18.8% ± 3.2%	5.8% ± 0.8%	**12.5% ± 2.4%**	11.3% ± 2.2%
Vemurafenib	**10.2% ± 1.2%**	1.3% ± 0.9%	5.4% ± 0.8%	5.1% ± 1.6%	13.3% ± 2.3%
Dabrafenib and Trametinib	9.7% ± 1.1%	2.1% ± 1.2%	**4.3% ± 0.7%**	**6.2% ± 1.7%**	5.5% ± 1.6%
Ipilimumab and Nivolumab	9.4% ± 1.1%	13.8% ± 2.9%	3.5% ± 0.6%	**9.7% ± 2.1%**	6% ± 1.6%
Nivolumab	8.7% ± 1.1%	13.6% ± 2.8%	**3.1% ± 0.6%**	11.5% ± 2.3%	4.4% ± 1.4%

*Note:* Bold values indicate where social media estimates overlap with a database.

**Table 4.** Second line treatment proportions and associated confidence intervals

Line 2 treatment	Flatiron drug proportion ± confidence interval	McKesson drug proportion ± confidence interval	MarketScan drug proportion ± confidence interval	PharMetrics+ drug proportion ± confidence interval	Social media drug proportion ± confidence interval
Pembrolizumab	**18.6% ± 2.5%**	35.8% ± 5.8%	8.1% ± 1.6%	23.9% ± 4.5%	16.4% ± 4.8%
Dabrafenib and Trametinib	13.7% ± 2.2%	1.9% ± 1.7%	**6.1% ± 1.4%**	**9.4% ± 3.1%**	6.6% ± 3.2%
Ipilimumab	13.7% ± 2.2%	6.9% ± 3.1%	9.1% ± 1.7%	**15% ± 3.8%**	24.3% ± 5.6%
Nivolumab	**11.2% ± 2.0%**	34.6% ± 5.8%	4.8% ± 1.3%	**12.7% ± 3.5%**	11.5% ± 4.2%
Ipilimumab and Nivolumab	5.8% ± 1.5%	**10% ± 3.6%**	3.2% ± 1%	3.8% ± 2%	8.4% ± 3.6%
Temozolomide	**5.3% ± 1.4%**	0.8% ± 1.1%	**4.6% ± 1.2%**	**4.4% ± 2.2%**	3.5% ± 2.4%

*Note:* Bold values indicate where social media estimates overlap with a database.

**Table 5.** Third line treatment proportions and associated confidence intervals

Line 3 treatment	Flatiron drug proportion ± confidence interval	McKesson drug proportion ± confidence interval	MarketScan drug proportion ± confidence interval	PharMetrics+ drug proportion ± confidence interval	Social media drug proportion ± confidence interval
Pembrolizumab	15.3% ± 3.9%	22.7% ± 10.1%	**4.1% ± 1.7%**	18.2% ± 6.1%	4.1% ± 4.6%
Dabrafenib and Trametinib	**12.9% ± 3.6%**	0% ± 0%	**7.0% ± 2.2%**	**13.0% ± 5.3%**	9.6% ± 6.8%
Nivolumab	**12.0% ± 3.5%**	**22.7% ± 10.1%**	5.3% ± 1.9%	**16.9% ± 5.9%**	19.2% ± 9.0%
Ipilimumab	**9.2% ± 3.1%**	**9.1% ± 6.9%**	7.0% ± 2.2%	**10.4% ± 4.8%**	16.4% ± 8.5%
Ipilimumab and Nivolumab	**8.9% ± 3.1%**	**18.2% ± 9.3%**	2.5% ± 1.4%	2.6% ± 2.5%	12.3% ± 7.5%
Temozolomide	**4.9% ± 2.3%**	0% ± 0%	**3.3% ± 1.5%**	5.2% ± 3.5%	1.4% ± 2.7%

*Note:* Bold values indicate where social media estimates overlap with a database.

For every treatment in every line, the social media treatment proportion estimate overlapped with at least one of the estimates coming from the databases. This is especially visible in the figures which display the data reported in the tables in graphical form.

## DISCUSSION

Here, we present an assessment of how treatment patterns in melanoma extracted from social media compare to treatment patterns obtained from more traditional EMR and claims databases.

We found that estimates obtained from social media overlapped with estimates from at least one of the databases, suggesting that certain social media represents a valid/alternative data source to understand treatment patterns in the real world.

When looking at overlap by treatment line, it appears that the higher the line number the greater the overlap. However, this needs to be seen in the context of uncertainty with confidence intervals becoming wider as sample size decreases (as seen in later lines of treatment).

It is notable that there is inherent variation in estimates obtained across the EMR and claims databases themselves, likely representing different patients and physicians (database coverage) and definitions of patients (by ICD coding or not). This is an interesting finding in itself and clearly highlights the bias present in observational studies generally. Whilst these databases may be viewed as a “gold standard” in outcomes research, the provenance of the data contained should always be considered. Given the variation between databases, we feel it is therefore more appropriate to compare the social media results to the range observed in all four databases rather than a single database in isolation as it is uncertain which database provides the least biased (most representative) result.

The fact that an automated process extracting data from publicly available patient forums where anyone can post somewhat aligns with data from databases perhaps may seem surprising, as it is known that there is a bias in the type of users that engage with their condition on social media (ie, posters on social media are perhaps likely to be a younger population).^2^ Indeed, the demographics of individuals posting on social media are rarely known and this will be a limitation when analytical strategies are attempted to mitigate biases in patient representativeness. Identifying proxies for demographic information is one potential solution for this and should be the subject for future work. Furthermore, there is also the issue of authenticity of posts, for example, we cannot determine if posters actually had the disease or if they were given the treatment described.^2^ One method for further validating posts, might include linking posts and EMR/claims data, however, this is prone to substantial logistical challenges itself. Nevertheless, based on this analysis there is no reason to expect that data extracted from social media cannot be used to understand treatment patterns, as an aspect of the patient experience, the use of which in general was recently encouraged by the Food and Drug Administration (FDA).^20^

### Limitations

There are a several limitations to this study. We used an automated processes to define patients, treatments taken and treatment sequencing in social media. These tools will not recognize the intricacies of human language such as sarcasm for example, which in turn may impact the validity of the results obtained. Further, as few users on these forums share information related to demographics (age, gender, and region), we were not able to consider where patients where posting from it is likely we have data from non-US patients in our social media analysis which would have influenced comparisons with US EMR and claims data.

With these caveats, our findings illustrate data from social media can provide another dimension to research on treatment patterns and an approach that is quick and with no access restrictions. More rigorous analytical methods can be applied for more specific questions (eg, patient perspectives on treatment received and effectiveness). However, it is clear from this study that social media offers significant potential as a valid real-world data source. Further work is needed to better understand patient perspectives of disease and treatment using these data.

## CODE AVAILABILITY STATEMENT

The code used for analysis will be shared upon request from the authors.

## DATA AVAILABILITY STATEMENT

The datasets analyzed during the current study are publicly available with websites cited for all data.

## CONTRIBUTIONS

S.R. is the guarantor of and conceived and designed the study. V.B. led the analysis. All contributed to the analysis and interpretation of the data. S.R. wrote the first draft and all contributed to subsequent drafts and the final paper. Flatiron Health did not participate in the data analysis presented in this manuscript.

## Funding

This study was supported by Bristol-Myers Squibb.

*Conflict of interest statement.* S.R., L.M., F.M., and B.M. are employees of Bristol Myers-Squibb. V.B. and V.S. are employees of Mu Sigma.

## Supplementary Material

ooz013_Supplementary_Tables
