# Supplementary material for: Validity of social media for assessing treatment patterns in oncology patients: a case study in melanoma
Source: JAMIA Open. 2019 Sep 3;2(4):416–22. doi: 10.1093/jamiaopen/ooz013 (PMC6994021; doi:10.1093/jamiaopen/ooz013)
Supplement: ooz013_Supplementary_Tables [file ooz013_supplementary_tables.docx]

**Supplementary Table 1**

**Melanoma treatment list used for post filtering.**

Italicised treatments were not considered as lines of treatment if received as monotherapy.

|  |
| --- |
| **Melanoma treatments** |
| Avelumab |
| Aldesleukin |
| Azacitidine |
| Bcg (Bacillus Calmette-Guerin) |
| Bendamustine Hydrochloride |
| Bevacizumab |
| Bicalutamide |
| Bortezomib |
| Cabazitaxel |
| Cabozantinib Malate |
| Capecitabine |
| Carboplatin |
| Carfilzomib |
| Carmustine |
| Cetuximab |
| Chlorambucil |
| Cisplatin |
| Cisplatin Vinblastine Dacarbazine |
| Cladribine |
| Cobimetinib |
| Crizotinib |
| Dabrafenib |
| Dacarbazine |
| Dasatinib |
| Decitabine |
| *Docetaxel* |
| Doxorubicin |
| Doxorubicin Pegylated Liposomal |
| Enzalutamide |
| Everolimus |
| Exemestane |
| Fludarabine Phosphate |
| Fluorouracil |
| Fulvestrant |
| Gemcitabine |
| Hydroxyurea |
| Ibrutinib |
| Idelalisib |
| Ifosfamide |
| Imatinib |
| *Interferon* |
| *Interferon Alfa-2A* |
| *Interferon Alfa-2B* |
| Ipilimumab |
| Irinotecan |
| Lenalidomide |
| Lomustine |
| Melphalan |
| Mercaptopurine |
| Methotrexate Sodium |
| Nivolumab |
| Obinutuzumab |
| Ofatumumab |
| Olaparib |
| Olaratumab |
| Oxaliplatin |
| Paclitaxel |
| Palbociclib |
| Panitumumab |
| *Peginterferon* |
| *Peginterferon Alfa-2A* |
| *Peginterferon Alfa-2B* |
| Pembrolizumab |
| Pertuzumab |
| Ramucirumab |
| Rigvir |
| Sunitinib Malate |
| T-Vec (Talimogene laherparepvec) |
| Tamoxifen |
| Tamoxifen Citrate |
| Temozolomide |
| Temsirolimus |
| Thalidomide |
| Tipiracil |
| Trametinib |
| Trifluridine |
| Vemurafenib |
| Vinblastine Sulfate |
| Vinorelbine Tartrate |

**Supplementary Table 2**

**Full melanoma treatment list dictionary used for post filtering including spelling mistakes, abbreviations and brand/generic names**

| Key | Value |
| --- | --- |
| Variant treatment mention | Corrected treatment mention |
| aldesleukin | Aldesleukin |
| il 2 | Aldesleukin |
| il2 | Aldesleukin |
| il-2 | Aldesleukin |
| leukin | Aldesleukin |
| interleukin-2 | Aldesleukin |
| interluken | Aldesleukin |
| proleukin | Aldesleukin |
| avelumab | Avelumab |
| bavencio | Avelumab |
| azacitidine | Azacitidine |
| azacitidine novaplus | Azacitidine |
| premierpro rx azacitidine | Azacitidine |
| vidaza | Azacitidine |
| bcg vaccine | Bcg |
| tice bcg | Bcg |
| tice bcg vaccine | Bcg |
| bendamustine | Bendamustine Hydrochloride |
| bendamustine hydrochloride | Bendamustine Hydrochloride |
| bendeka | Bendamustine Hydrochloride |
| treanda | Bendamustine Hydrochloride |
| avastin | Bevacizumab |
| bevacizumab | Bevacizumab |
| bicalutamide | Bicalutamide |
| casodex | Bicalutamide |
| bortezomib | Bortezomib |
| velcade | Bortezomib |
| cabazitaxel | Cabazitaxel |
| jevtana | Cabazitaxel |
| cabometyx | Cabozantinib Malate |
| cabozantinib | Cabozantinib Malate |
| cabozantinib malate | Cabozantinib Malate |
| cometriq | Cabozantinib Malate |
| capecitabine | Capecitabine |
| xeloda | Capecitabine |
| amerinet choice carboplatin | Carboplatin |
| carboplatin | Carboplatin |
| carboplatin n+ novaplus | Carboplatin |
| carboplatin novaplus | Carboplatin |
| carboplatin otn | Carboplatin |
| paraplatin | Carboplatin |
| paraplatin novaplus | Carboplatin |
| premierpro rx carboplatin | Carboplatin |
| carfilzomib | Carfilzomib |
| kyprolis | Carfilzomib |
| bicnu | Carmustine |
| carmustine | Carmustine |
| gliadel | Carmustine |
| cetuximab | Cetuximab |
| erbitux | Cetuximab |
| chlorambucil | Chlorambucil |
| leukeran | Chlorambucil |
| cisplatin | Cisplatin |
| cisplatin amerinet choice | Cisplatin |
| cisplatin novaplus | Cisplatin |
| platinol | Cisplatin |
| platinol-aq | Cisplatin |
| platinol-aq novaplus | Cisplatin |
| cvd | Cisplatin-Vinblastine-Dacarbazine |
| cladribine | Cladribine |
| cladribine novaplus | Cladribine |
| leustatin | Cladribine |
| cobimetinib | Cobimetinib |
| cotellic | Cobimetinib |
| crizotinib | Crizotinib |
| xalkori | Crizotinib |
| dabrafenib | Dabrafenib |
| dabrafenib mesylate | Dabrafenib |
| taf | Dabrafenib |
| tafinlar | Dabrafenib |
| dacarbazine | Dacarbazine |
| dacarbazine novaplus | Dacarbazine |
| dtic-dome | Dacarbazine |
| dasatinib | Dasatinib |
| sprycel | Dasatinib |
| dacogen | Decitabine |
| decitabine | Decitabine |
| decitabine novaplus | Decitabine |
| premierpro rx decitabine | Decitabine |
| docetaxel | Docetaxel |
| taxotere | Docetaxel |
| doxil | Doxorubicin |
| doxorubicin pegylated liposomal | Doxorubicin Pegylated Liposomal |
| enzalutamide | Enzalutamide |
| xtandi | Enzalutamide |
| afinitor | Everolimus |
| afinitor disperz | Everolimus |
| everolimus | Everolimus |
| zortress | Everolimus |
| aromasin | Exemestane |
| exemestane | Exemestane |
| fludara | Fludarabine Phosphate |
| fludarabine | Fludarabine Phosphate |
| fludarabine phosphate | Fludarabine Phosphate |
| fludarabine phosphate novaplus | Fludarabine Phosphate |
| oforta | Fludarabine Phosphate |
| otn fludarabine phosphate | Fludarabine Phosphate |
| 5-fluorouracil | Fluorouracil |
| adrucil | Fluorouracil |
| carac | Fluorouracil |
| efudex | Fluorouracil |
| efudex occlusion pack | Fluorouracil |
| fluoroplex | Fluorouracil |
| fluorouracil | Fluorouracil |
| fluorouracil novaplus | Fluorouracil |
| premierpro rx fluorouracil | Fluorouracil |
| tolak | Fluorouracil |
| faslodex | Fulvestrant |
| fulvestrant | Fulvestrant |
| gemcitabine | Gemcitabine |
| gemcitabine hcl | Gemcitabine |
| gemcitabine hydrochloride | Gemcitabine |
| gemcitabine novaplus | Gemcitabine |
| gemzar | Gemcitabine |
| ifex/mesnex | Gemcitabine |
| ifex/mesnex novaplus | Gemcitabine |
| premierpro rx gemcitabine | Gemcitabine |
| droxia | Hydroxyurea |
| hydrea | Hydroxyurea |
| hydroxycarbamide | Hydroxyurea |
| hydroxyurea | Hydroxyurea |
| mylocel | Hydroxyurea |
| ibrutinib | Ibrutinib |
| imbruvica | Ibrutinib |
| idelalisib | Idelalisib |
| zydelig | Idelalisib |
| ifex | Ifosfamide |
| ifex novaplus | Ifosfamide |
| ifosfamide | Ifosfamide |
| ifosfamide novaplus | Ifosfamide |
| gleevec | Imatinib |
| imatinib | Imatinib |
| imatinib mesylate | Imatinib |
| interferon | Interferon |
| interferon 2 | Interferon |
| interferons | Interferon |
| interferron | Interferon |
| interfuron | Interferon |
| intron | Interferon |
| interferon alfa-2a | Interferon Alfa-2A |
| roferon | Interferon Alfa-2A |
| roferon-a | Interferon Alfa-2A |
| interferon alfa 2b | Interferon Alfa-2B |
| interferon alfa-2b | Interferon Alfa-2B |
| intron a | Interferon Alfa-2B |
| rebetron 1000 | Interferon Alfa-2B |
| rebetron 1200 | Interferon Alfa-2B |
| rebetron 600 | Interferon Alfa-2B |
| ipilimumab | Ipilimumab |
| ippi | Ipilimumab |
| lpilumamab | Ipilimumab |
| rervoy | Ipilimumab |
| vervoy | Ipilimumab |
| yarvoy | Ipilimumab |
| yervoy | Ipilimumab |
| yrvy | Ipilimumab |
| yevoy | Ipilimumab |
| amerinet choice irinotecan hydrochloride | Irinotecan |
| camptosar | Irinotecan |
| camptosar novaplus | Irinotecan |
| irinotecan | Irinotecan |
| irinotecan hcl novaplus | Irinotecan |
| irinotecan hydrochloride | Irinotecan |
| irinotecan liposome | Irinotecan |
| onivyde | Irinotecan |
| premierpro rx irinotecan hcl | Irinotecan |
| lenalidomide | Lenalidomide |
| revlimid | Lenalidomide |
| ceenu | Lomustine |
| gleostine | Lomustine |
| lomustine | Lomustine |
| alkerlan | Melphalan |
| mercaptopurine | Mercaptopurine |
| mercaptopurine monohydrate | Mercaptopurine |
| purinethol | Mercaptopurine |
| purixan | Mercaptopurine |
| abitrexate | Methotrexate Sodium |
| folex | Methotrexate Sodium |
| folex pfs | Methotrexate Sodium |
| methotrexate | Methotrexate Sodium |
| methotrexate lpf sodium | Methotrexate Sodium |
| methotrexate novaplus | Methotrexate Sodium |
| methotrexate sodium | Methotrexate Sodium |
| mexate | Methotrexate Sodium |
| mexate-aq | Methotrexate Sodium |
| otrexup | Methotrexate Sodium |
| premierpro rx methotrexate | Methotrexate Sodium |
| rasuvo | Methotrexate Sodium |
| rheumatrex dose pack | Methotrexate Sodium |
| trexall | Methotrexate Sodium |
| xatmep | Methotrexate Sodium |
| nivolumaab | Nivolumab |
| nivolumab | Nivolumab |
| nivulmab | Nivolumab |
| nivolumad | Nivolumab |
| opdivo | Nivolumab |
| opidivo | Nivolumab |
| gazyva | Obinutuzumab |
| obinutuzumab | Obinutuzumab |
| arzerra | Ofatumumab |
| ofatumumab | Ofatumumab |
| lynparza | Olaparib |
| olaparib | Olaparib |
| lartruvo | Olaratumab |
| olaratumab | Olaratumab |
| eloxatin | Oxaliplatin |
| oxaliplatin | Oxaliplatin |
| oxaliplatin novaplus | Oxaliplatin |
| premierpro rx oxaliplatin | Oxaliplatin |
| abraxane | Paclitaxel |
| nov-onxol | Paclitaxel |
| onxol | Paclitaxel |
| paclitaxel | Paclitaxel |
| paclitaxel amerinet choice | Paclitaxel |
| paclitaxel novaplus | Paclitaxel |
| paclitaxel otn | Paclitaxel |
| paclitaxel protein-bound | Paclitaxel |
| premierpro rx paclitaxel | Paclitaxel |
| taxol | Paclitaxel |
| taxol novaplus | Paclitaxel |
| ibrance | Palbociclib |
| palbociclib | Palbociclib |
| panitumumab | Panitumumab |
| vectibix | Panitumumab |
| peginterferon | Peginterferon |
| pegasys | Peginterferon Alfa-2A |
| pegasys proclick | Peginterferon Alfa-2A |
| peginterferon alfa-2a | Peginterferon Alfa-2A |
| peg intron rp | Peginterferon Alfa-2B |
| peginterferon alfa-2b | Peginterferon Alfa-2B |
| pegintron | Peginterferon Alfa-2B |
| sylatron | Peginterferon Alfa-2B |
| kaytruda | Pembrolizumab |
| keytruda | Pembrolizumab |
| keytrude | Pembrolizumab |
| keytryda | Pembrolizumab |
| ktruda | Pembrolizumab |
| pembro | Pembrolizumab |
| pembrolizumab | Pembrolizumab |
| perjeta | Pertuzumab |
| pertuzumab | Pertuzumab |
| cyramza | Ramucirumab |
| ramucirumab | Ramucirumab |
| rigvir | Rigvir |
| sunitinib | Sunitinib Malate |
| sunitinib malate | Sunitinib Malate |
| sutent | Sunitinib Malate |
| tamoxifen | Tamoxifen |
| nolvadex | Tamoxifen Citrate |
| soltamox | Tamoxifen Citrate |
| tamoxifen citrate | Tamoxifen Citrate |
| temodar | Temozolomide |
| temozolomide | Temozolomide |
| tremador | Temozolomide |
| temsirolimus | Temsirolimus |
| torisel | Temsirolimus |
| thalidomide | Thalidomide |
| thalomid | Thalidomide |
| tipiracil | Tipiracil |
| mekinist | Trametinib |
| trametinib | Trametinib |
| trametinib dimethyl sulfoxide | Trametinib |
| trifluridine | Trifluridine |
| imlygic | T-Vec |
| t-vec | T-Vec |
| vem | Vemurafenib |
| vemurafenib | Vemurafenib |
| zelboraf | Vemurafenib |
| zelboref | Vemurafenib |
| zelborif | Vemurafenib |
| zelbraf | Vemurafenib |
| velban | Vinblastine Sulfate |
| velsar | Vinblastine Sulfate |
| vinblastine | Vinblastine Sulfate |
| vinblastine sulfate | Vinblastine Sulfate |
| amerinet choice vinorelbine tartrate | Vinorelbine Tartrate |
| navelbine | Vinorelbine Tartrate |
| vinorelbine | Vinorelbine Tartrate |
| vinorelbine novaplus | Vinorelbine Tartrate |
| vinorelbine tartrate | Vinorelbine Tartrate |
| ipi | Ipilimumab |
| nivo | Nivolumab |
| Z ( with space on both sides ) | Vemurafenib |
| zelbarof | Vemurafenib |
| zel | Vemurafenib |
| divo | Nivolumab |
| odivo | Nivolumab |
| Dafrafenib | Dabrafenib |
| MK-375 | Pembrolizumab |
| zebraf | Vemurafenib |
| temadar | Temozolomide |
| HD-Interferon | Interferon |
| meknist | Trametinib |
| Ippy | Ipilimumab |
| novo | Nivolumab |
| vemurafinib | Vemurafenib |
| mekenist | Trametinib |
| optivo | Nivolumab |
| zelbraf | Vemurafenib |
